# Would Asexualization of Chronic Criminals, Sexual Perverts and Hereditary Defectives Benefit Society and Elevate the Human Race?

**Published:** 1896-11

**Authors:** E. Stuver

**Affiliations:** Rawlins, Wyoming


### TEXAS MEDICAL JOURNAL.

ESTABLISHED JULY, 1885.

PUBLISHED MONTHLY.—SUBSCRIPTION $2.00 A YEAR.

VOL. XII. AUSTIN, NOVEMBER, 1896. NO. 5.

### Original Contributions.

For the Texas Medical Journal.

### WOULD ASEXUALIZATION OF CHRONIC CHRIMINALS, SEXUAL PERVERTS AND HEREDITARY DEFECTIVES BENEFIT SOCIETY AND ELEVATE THE HUMAN RACE?

BY E. STUVER, M. D., PH. D., RAWLINS, WYOMING.

"TO SUBJECT the human race to the same laws that stockmen apply to their cattle in eliminating the poorer breeds, and castrate those who are incurably diseased, together with lunatics, sexual perverts, thieves and criminals, would I think, if practicable, be retrogressive and unworthy of the soaring aspirations of scientific researches, which can accomplish no possible good that is not humanitarian in its application."

I must confess that when I read the above and opening sentence of the letter of Dr. De Causey, published in the April, 1896 issue of the TEXAS MEDICAL JOURNAL, I was considerably surprised. His summary method of disposing of a prominent part of a question, that at the present time is engaging the earnest thought and painstaking investigations of many of the greatest scientists and philanthropists of the age; viz: the proper treatment and disposition of the criminal and defective classes, is certainly an easy solution of the question, provided we are strong enough believers in the doctrine of *Laissez faire* to arrive at the conclusion that the matter does not concern us, and that the better policy is to let things drift regardless of the consequences such a course might entail.

Looking backward through the vistas of the past to the earliest historical times; yea far into the dim twilight of tradition, we see passing before us a stately procession of physicians, who not only ministered to the sufferings of the age in which they lived, but were ever in the front rank in all efforts and undertakings to prevent the ravages of disease and death, as well as to raise mankind to a higher, physical and moral plane. With such examples of self-sacrificing devotion to truth, and consecration to the cause of humanity in the past to direct and stimulate us; with such vital and momentous questions of the present, as are staring us in the face, and like Banquo's ghost, will not down, but demand settlement and solution, I believe it is the duty of every patriotic citizen, and especially of every physician, whose training and opportunities give him a broader and more intimate knowledge of all physical, intellectual, and I might say moral deviations or perversions which are insidiously sapping the foundations of our national greatness and endangering our future progress and happiness, to exert every effort in his power, in assisting to neutralize or remove these malign agencies and tendencies to degeneration which are so prevalent and potent at the present time.

Taking this view of the matter, I regard the statement that asexualization, as a remedy for these evils, is not in accordance with humanitarian and scientific aspirations, as singularly unfortunate.

While all will admit that man is the highest and noblest product of organic evolution, still I think few competent scientists would have the temerity to claim that he is superior to and can, with impunity, disregard the laws governing the physical integrity and well being of other divisions of the animal kingdom. If this is true, the statement, that "to subject the human race to the same laws that stockmen apply to their cattle . . . . . is retrogressive," is not only an erroneous but highly dangerous doctrine.

As is very well known, the more highly differentiated an organism is, the more unstable it becomes; so that the charming accomplishments and graces that adorn our civilization are the qualities first lost when decay of any kind attacks the organism.

It is a serious reflection on the good sense and intelligence of a people that they should exert the greatest care and expense in securing the best breeds of horses, cattle, and all kinds of domestic animals, but scarcely give a thought, save possibly a mercenary one, to the endowments of the life partners of their children. How often are the pure and innocent married to diseased and degraded wrecks of humanity, whose only recommendation is that they possess great wealth, or occupy so-called high social positions in the world!

Such unions as these pollute the very sources of being, entailing disease, suffering and sorrow, and leaving a devastating trail through many generations to come. Every physician of experience knows that this picture is not overdrawn, and that the diseased condition thus transmitted not only lowers the physical, mental and moral powers, but by contaminating the blood—the very source of all vital energy—creates an instability of the nervous centres, thereby depriving their possessors of that strength and mental poise which would enable them to resist the many temptations and unfavorable conditions of their environment, and is largely responsible for that nervous erethism which leads to sexual, alcoholic, and all sorts of excesses which make their unfortunate victims entirely irresponsible for their conduct in many instances. Such persons are diseased, and should be treated accordingly. They should be removed entirely from the jurisdiction of Judge *Lynch*, and confided to the care of thoroughly competent physicians who should have ample authority to adopt such measures as will tend to the relief of the sufferer and the protection of society.

If there is any truth in our much vaunted principle, that laws should be enacted for the "greatest good of the greatest number," I believe it ought to be carried into effect in a practicable and rational manner, and that nature's plan of making the punishment consequent upon, and naturally and inevitably follow the offense, should be carried out.

Under such a plan, those suffering from incurable and transmissible diseased would not be permitted to marry, and syphilitics and others dangerous to the public health, would be isolated or confined in a hospital, and not permitted to leave until they were pronounced cured by competent authority, and all danger of communicating disease had passed.

I believe every fair-minded and thinking physician will admit that such a plan would be of immense benefit to humanity, and at once greatly limit the prevalence of disease and its attendant companion, crime.

If it is legitimate and right for society, in order to protect itself, to limit the privileges of the individual, and even in extreme cases to carry this limitation to the deprivation of life, I can see no reason why it is not rational, right and *humane* to adopt methods that will strike at the root of the whole matter and limit the perpetuation of the diseased, vicious and criminal classes.

It is a well-established principle in medical practice that whenever the predisposing or exciting causes of diseases can be ascertained, that they should be removed as promptly as possible. If a piece of bone or a blood clot is causing pressure on the brain, and endangering the life of the individual, the careful surgeon will try to remove the cause; if a kidney is irreparably diseased, or an arm or leg gangrenous, he will remove the damaged organ or member, and try to save the life of the patient, even though seriously mutilated; if a tight or adherent prepuce is causing reflex irritation, or an injured eye threatens the integrity of its fellow, he does not hesitate to sacrifice the offending part. When a person becomes insane, he is at once shut up in an asylum, and even the poor old irresponsible drunkard can look back and recall numerous sentences to the "Island," or some other place of detention; but when it comes to asexualizing chronic criminals, sexual perverts, *et id omne genus*, who are not only injuring themselves, but are a constant menace to society, both present and future, we are met by a storm of opposition; we are told that it is an invasion of individual liberty; that it is "unworthy of the soaring aspirations of scientific researches," and that "the idea is revolting to the instincts of humanity." Such sentiments as these are, in my opinion, sustained neither by the principles of justice or logic, and I believe it is the duty of every physician to be a leader in all movements tending to the suppression of disease, vice and crime, and to make a manful stand for truth, right and the betterment of mankind, even if so-called popular opinion may not sustain his position at the time.

All great reforms move slowly, and not only that, but require much self-sacrificing devotion and heroism to carry them into effect. While this is true, still if the vast army of physicians throughout the land would insist that the incurably diseased should not be permitted to marry and thus transmit their defects; that those suffering from contagious disorders should be placed under strict surveilance or isolated, and that certain classes of chronic confirmed, or hereditary criminals and sexual perverts, should be asexualized or deprived of the power to beget a progeny endowed with all their propensities, and that, too, in an accentuated form, public opinion would soon sustain their demands, and the necessary laws be enacted. All history and science teach us that neither individuals nor nations can remain stationary; they must either advance to a higher development and a nobler civilization or, by a retrograde process, degenerate, decay and disappear.

Our wonderful progress and remarkable material achievements as a nation, have, I fear, made us unduly optimistic, and either closed our eyes to, or rendered us careless of the many demoralizing tendencies that are sapping the vitality of our people and will, unless overcome, endanger the integrity and greatness of our nation. No nation or people can neglect the cultivation of those virtues that go to make up a high and noble character, and long survive. All history proves that as long as nations maintained these high ideals, they were invincible, but as soon as they gave themselves up to luxury, idleness, sensuality and vice, in all its varied forms, they became an easy prey to surrounding barbarian hordes; indeed, in the absence of outside enemies, would probably have fallen to pieces from their own inherent weakness and depravity.

That there are tendencies to degeneration, and some very strong ones, too, in our present advanced civilization, no one competent to form an intelligent opinion will, I believe, deny. Whether these tendencies will become *dominant* and lead to our downfall, the future alone can reveal. Dr. Max Nordau, in his work, "Degeneration," has clearly shown their influence on the literary and artistic productions of the age. While this work may be overdrawn, and some of his characters painted in too vivid colors, still, notwithstanding the virulent attacks of his critics, who have heaped all sorts of abuse upon him, every unprejudiced person who has anything like a comprehensive general knowledge of the literature of the last twenty years must admit that Nordau's work contains a great deal of truth and a wide scientific and literary knowledge.

To any one who desires to know what the author really claims, I would say, read "Degeneration" itself, rather than the criticisms of some of the "O Lord, I thank Thee, I am not like other men" style of critics who have assailed his conclusions. You will find much to admire, many things to approve, and while you may not always accept his conclusions, you will admit that he "holds the mirror up to nature" and reveals many things which should be widely known, and that he is by no means the destructive pessimist that he has been claimed to be. If Nordau had rendered no other service to mankind than calling attention to the vileness and demoralizing influence of much of our so-called realistic literature, he would be entitled to the gratitude of every lover of decency.

Moral excellence and virtue are of the greatest importance to a nation. If we would avert the fate of Rome, we must avoid the vices which led to her downfall. In illustrating the evil effects of a lowered national morality, the learned and brilliant Dr. James T. Whittaker (see *Journal of American Medical Association*, April 11, 1896, p. 700) speaks as follows: "It is never better to compromise with that kind of evil. It is better to starve than to steal, and it is better to die of disease engendered by drinking excrement than to countenance bribery in any form. Physical is never so bad as moral corruption. All history shows that though a plague may desolate, it is immorality alone that can destroy a nation. Rome fell, not on account of the plagues, and not on account of the Goths and the Vandals, but because of the failure in the crop of Roman children."

The way to promote physical, intellectual and moral purity and integrity is to stamp out opposing tendencies, and if that cannot be done, to inhibit them as much as possible.

It has been very clearly shown by Dr. F. E. Daniel (who, by the way, was one of the pioneers in this field), see TEXAS MEDICAL JOURNAL, December, 1893, and also May, 1896; Dr. Opheus Evarts, of Cincinnati, Ohio; Dr. Robert Boal, of Lacon, Ill. (see *Journal of American Medical Association*, Sept. 15, 1895), and others, that asexualization would furnish us the very best and strongest inhibitive, protective and curative means for dealing with certain classes of criminals and sexual perverts.

In a paper read before the Colorado State Medical Association, June, 1895, I gave a general outline of the subject, and will here state the conclusions arrived at in that paper, viz.:

"1st. By removing or modifying their exciting causes and favoring circumstances, it (asexualization) would greatly limit the dissemination of disease, vice and crime.

"2nd. It would furnish a humane and rational punishment for a large and continually increasing number of criminals, and satisfy the demands of justice.

"3rd. Owing to the innate repugnance toward this condition, it would be a powerful deterrent, and thereby limit the prevalence of crime, and protect society against the criminal.

"4th. By removing the procreative capacity of defectives and criminals, it would do more to diminish their numbers, and protect society against their ravages, while at the same time raising the physical, intellectual and moral standard of the race than all other punishments combined.

"5th. It would be the humane and rational method of treating these classes, because it would protect them against harsh and brutal punishments, and by removing *the cause* of their obliquities, transform many of them into useful and productive citizens."

---

For the Texas Medical Journal.

